# WD40 repeat 43 mediates cell survival, proliferation, migration and invasion via vimentin in colorectal cancer

**DOI:** 10.1186/s12935-021-02109-1

**Published:** 2021-08-09

**Authors:** Zijian Li, Min Feng, Jie Zhang, Xingzhou Wang, En Xu, Chao Wang, Fengcen Lin, Zhi Yang, Heng Yu, Wenxian Guan, Hao Wang

**Affiliations:** 1 Department of General Surgery, Drum Tower Clinical Medical College of Nanjing Medical University, Nanjing, 210008 Jiangsu People’s Republic of China; 2 Department of General Surgery, Affiliated Drum Tower Hospital, Medical School of Nanjing University, Nanjing, 210008 Jiangsu People’s Republic of China

**Keywords:** WDR43, VIM, CRC, Biomarker

## Abstract

**Background:**

WD40 repeat (WDR)43 is an RNA-binding protein that belongs to the WDR domain protein family. Its biological function is largely unclear, particularly in colorectal cancer (CRC).

**Methods:**

In the present study, we searched the TCGA database and found the correlation between WDR43 and CRC. Subsequently, the high expression of WDR43 in human clinical samples of CRC was validated and we further examined the biological functions of it in CRC cells. Finally, we explored potential downstream proteins or pathways and established subcutaneous xenograft model to verify our findings.

**Results:**

Immunohistochemistry of 16 patient specimens confirmed that the expression of WDR43 was elevated in CRC. WDR43 knockdown was shown to increase apoptosis and inhibit the proliferation, migration and invasion of CRC cells in vitro and reduce tumorigenesis in animal models. In addition, it was found that WDR43 knockdown inhibited vimentin (VIM) expression in CRC cells and overexpression of VIM can partially reverse the effects of WDR43 both in vitro and in vivo.

**Conclusion:**

In conclusion, the role of WDR43 in the occurrence and development of CRC was investigated in the present study. WDR43 may serve as a valuable biomarker and provide new options for the diagnosis and treatment of colorectal cancer.

**Supplementary Information:**

The online version contains supplementary material available at 10.1186/s12935-021-02109-1.

## Introduction

Colorectal cancer (CRC) accounts for 10% of the total cases of cancer and cancer-related mortality worldwide per annum [1]. Particularly in developing countries, the incidence of CRC is gradually increasing [2]. In China, despite the widespread use of early screening for individuals with a high-risk family history, most patients are diagnosed at the late stage of the disease and cannot benefit from surgical treatment [3]. Patients with distant metastases generally have a relatively poor prognosis, due to accelerated tumor spreading and resistance to chemotherapy and radiotherapy, which makes CRC a difficult clinical challenge [4, 5]. Therefore, it is very important to identify novel targets for the treatment and diagnosis of CRC. The WD40 repeat (WDR) domain is a domain composed of multiple β-helical structures. It is usually used as a scaffold for protein interaction and provides a platform for the assembly of multi-protein complexes [6]. Proteins containing the WDR domain participate in a wide range of cellular networks, such as signal transduction, transcriptional regulation, cell cycle control, cytoskeleton assembly and chromatin modification, many of which are associated with human diseases [7]. The WDR43 protein belongs to the protein family containing the WDR domain. It is usually involved in the assembly of small-subunit processome as a conserved component, and mediates the transcription and processing of small-subunit ribosomal RNA (18S rRNA), which plays a key role in the biogenesis of ribosomes [8, 9]. Studies have shown that WDR43 can suspend the release of Pol II and promote transcription extension to regulate the high-level transcription and translation process in the pluripotency of embryonic stem cells [10]. WDR43 mutations were not only found to cause defects in craniofacial development in zebrafish [11], but were also associated with the occurrence of human estrogen receptor-negative breast cancer [12]. These findings suggested that WDR43 plays a key role in cell proliferation and may be involved in the occurrence and development of cancer. However, the specific role of WDR43 in CRC remains unclear; therefore, the present study was designed to explore the impact of WDR43 on the biological behavior of CRC and determine whether it may be of value as a new target for the diagnosis and treatment of CRC.

## Materials and methods

### Immunochemistry

Colorectal multi-tumor tissue microarrays (TMAs) were purchased from Genechem Co., Ltd. The TMAs were baked in an incubator at 60 °C for 30 min and then immersed in xylene for 10 min. Soaked them in 95%, 85% and 75% alcohol for 10 min and blocked with fresh 3% H_2_O_2_. Heat 0.01 M sodium citrate buffer solution (pH 6.0) in microwave oven on high heat to boil, and put the TMAs in on low heat for 20 min. The TMAs were then blocked with 5% goat serum dissolved in PBS for 60 min and incubated with Rabbit polyclonal anti-WDR43 antibody (cat no. ab174906; dilution, 1:50; Abcam) overnight at 4 °C. Added the secondary antibody and incubated at room temperature for 60 min. After coloring for 5–10 min by DAB, stained again with hematoxylin. Following staining, the samples were assessed by a senior pathologist, and a blinded manner were taken to record the immunohistochemical results. The final score of each microarray was based on the proportion of positively colored cells (0, 0–5%; 1, 6–25%; 2, 26–50%; 3, 51–75%; and 4, 76–100%) and staining intensity (0, no staining; 1, thin staining; 2, middling staining; and 3, strong staining). Low expression was defined as a final score of < 6, and high as a final score of ≥ 6. The present study was authorized by the Ethics Committee of the Nanjing Drum Tower Hospital.

### Cell culture

The DLD-1 CRC cell line was purchased from Procell Life Science & Technology Co., Ltd., and the RKO, SW480, NCM460 and HCT116 cell line from Nanjing KeyGen Biotech Co., Ltd. All cell lines were STR-authenticated. These cells were incubated at 37 °C, 5% CO_2_, McCoy’s 5A or DMEM medium (Thermo Fisher Scientific, Inc.) containing 10% fetal bovine serum (Gibco, USA) for HCT116 cells and the rest cell lines, respectively. The medium was renewed every 3 days and the cells were passaged when the cell density reached 80–90%.

### Lentiviral transfection

The shRNA and vimentin overexpressed plasmid vectors were constructed for lentiviral (GeneChem, Inc.) infection to stably knockdown WDR43 and overexpress VIM (Additional file 1). The whole transfection procedure was then performed following the manufacturer’s instructions. Puromycin was used to eliminate the untransfected cells 3 days after transfection. The transfection of the surviving cells was confirmed by RT-qPCR. Following is the shRNA sequences of WDR43 knockdown: CGATGAACCTGTCTATATT. The source of the VIM overexpression construct is showed in Additional file 10.

### RNA extraction and RT-qPCR

Total RNA of cells was extracted using TRIzol^®^ reagent (Merck KGaA) and detected by BioDrop μLITE + (Biochrom, Ltd.). RT was then performed using HiScript III RT SuperMix for qPCR (Vazyme Biotech Co., Ltd.) to synthesize cDNA. Next, RT-qPCR was executed on a ViiA™ 7 Real-Time PCR System (Thermo Fisher Scientific, Inc.) using the comparative threshold cycle (2-ΔΔCt) method. Target gene expression at the mRNA level was relatively quantified using GAPDH as the internal reference gene. Following is primer sequences: WDR43, 5′-CCAGGGCTTAGAAAGTAACGA-3′ forward and 5′-TCAGGCAACGTGGACAGGTAT-3′ reverse (final product size, 228 bp); GAPDH, 5′-TGACTTCAACAGCGACACCCA-3′ forward and, 5′-CACCCTGTTGCTGTAGCCAAA-3′ reverse (final product size, 121 bp). Acquired data were finally analyzed by GraphPad Prism 7.0 (GraphPad Software, Inc.).

### Protein extraction and immunoblotting

Proteins were extracted from cells with 100 μl RIPA buffer containing 1:100 Complete™ Protease Inhibitor Cocktail (Roche Diagnostics). Following lysing and centrifugation, the protein-containing supernatant was mixed with 5X SDS-PAGE loading buffer (1:4 ratio; Biosharp Life Sciences) and boiled for 5 min to denaturate the protein. BCA assay (Nanjing KeyGen Biotech Co., Ltd.) was used for protein quantification. Equal amounts of proteins (15 μg) from each sample were loaded in 10% gel (PG112, epizyme) and transferred on PVDF membrane (1000021, GE Healthcare). The membranes were then cut into stripes and blocked with 10% skim milk (1172GR500, Biofroxx). Then the stripes were incubated with primary antibodies including anti-WDR43, anti-N-cadherin, anti-E-cadherin, anti-Slug, anti-Snail, anti-VIM, anti-MYC, anti-FN1, anti-MMP2, anti-(p-)β-catenin, anti-(p-)ERK1/2, anti-(p-)P-38, anti-(p-)Akt, anti-(p-)NF-κB p65, anti-(p-)mTOR(all from abcam, dilution, 1:1000) and the internal reference protein GAPDH (Cat. No. sc-32233; dilution, 1:2000), followed by incubation with secondary antibodies for two hours at room temperature (7074S, dilution, 1:4000, Cell-Signaling Technology, Inc.). 20X LumiGLO^®^ Reagent and 20X peroxide (7003S; Cell-Signaling Technology, Inc.) were used in development following the instruction, and the bands were imaged using a Tanon 5200 Chemiluminescent Imaging System (Tanon Science & Technology Co., Ltd).

### MTT and Celigo assay

Cells that had received different treatments were harvested using trypsin with 0.25% EDTA (Thermo Fisher Scientific, Inc.) and counted using Countstar^®^ BioTech Automated Cell Counter (Countstar). The cells were then seeded into 96-well plates (1.5 × 103 cells/well). For Celigo assays, the number of cells was counted each day using the Celigo Image Cytometer (Nexcelom Bioscience LLC). For MTT assays, the cells were then incubated, respectively, for 24, 48, 72, 96 and 120 h. MTT reagent (20 µl; 5 mg/ml; Genview) was added 4 h before the incubation was terminated. Once precipitates became visible, the supernatants were carefully discarded and 100 µl dimethyl sulfoxide was added for 2–5 min to dissolve the precipitates. The absorbance value of each well was detected by a microplate reader at 490 nm. Each experiment was repeated at least three times.

### Colony formation assay

Transfected cells were cultivated into each well of a 6-well culture plate (1.5 × 103 cells/well) and cultured for 8 days, with renewal of the medium every 3 days during the incubation. The cells were then washed with 1 ml PBS at a time, fixed by 1 ml 4% paraformaldehyde for 25 min at room temperature, and rewashed once with 1 ml PBS. Then, cells were colored with 1 ml crystal violet solution (Beyotime, C0121) (Sangon Biotech Co., Ltd.) for 15 min at room temperature and then washed with ddH_2_O several times. The plate was air-dried and photographed with a digital camera to count the number of colonies containing > 50 cells. Following formula was used to calculate the colony formation efficiency: Colony formation efficiency = (number of colonies/number of inoculated cells) × 100%.

### Flow cytometry

Flow cytometry was used to analyze the apoptotic rate of tumor cells that had received different treatments. Annexin V Apoptosis Detection Kit II (556570; BD Biosciences) was used for incubation with the collected cells to detect the apoptotic rate. The results were detected and obtained by BD AccuriTM C6 Plus cell analyzers (BD Biosciences). FlowJo 10.4 (FlowJo, LLC) was used to analyze the final results.

### Cell migration and invasion

To evaluate the effect of WDR43 on migration and invasion of cells, Transwell assays were performed. Briefly, Transwell^®^ chambers with an 8.0-µm Pore Polycarbonate Membrane (Cat. No. 3422; Corning) were placed in a 24-well plate, with serum-free culture medium in the upper chamber and medium having 10% fetal bovine serum in the lower chamber; tumor cells (1 × 105 cells) were placed into the upper chamber. Following 24 h of cultivation, 4% paraformaldehyde was used to fix the cells for 15 min and then cells were colored with crystal violet solution (Beyotime, C0121) for 20 min. Following washing with PBS and wiping, the final results were photographed under Inverted Microscope Solution DMi8 S Platform (Leica Microsystems, Inc.) and analyzed by Leica Application Suite X (Leica Microsystems, Inc.). For cell invasion assays, the chambers were coated with Corning^®^ Matrigel^®^ Basement Membrane Matrix (cat. no. 356324; Corning) and incubated for 30 min; the rest of the procedure was coincident as that for the migration assay.

### In vivo experiments

Male BALB/c nude mice aged 4–6 weeks were provided by the Nanjing Medical University. Tumor cells were digested with trypsin and adjusted to 1 × 10^7^ cells/ml, and then subcutaneously injected into the back of mice. Each group contained a minimum of five mice. Mice were sacrificed 4 weeks after treatment, and tumors were subsequently resected to assess weight and volume.

### Statistical analysis

All experiments were repeated at least three times. GraphPad Prism 7.0 (GraphPad Software) was used to analyze the data and determine the significance of the differences. For categorical data, student *t* test was performed for comparisons between two groups, and one-way ANOVA for comparisons among multiple groups. *p* < 0.05 was considered statistically significant.

## Results

### WDR43 is associated with the stage and tissue subtypes of CRC

**Fig. 1:**
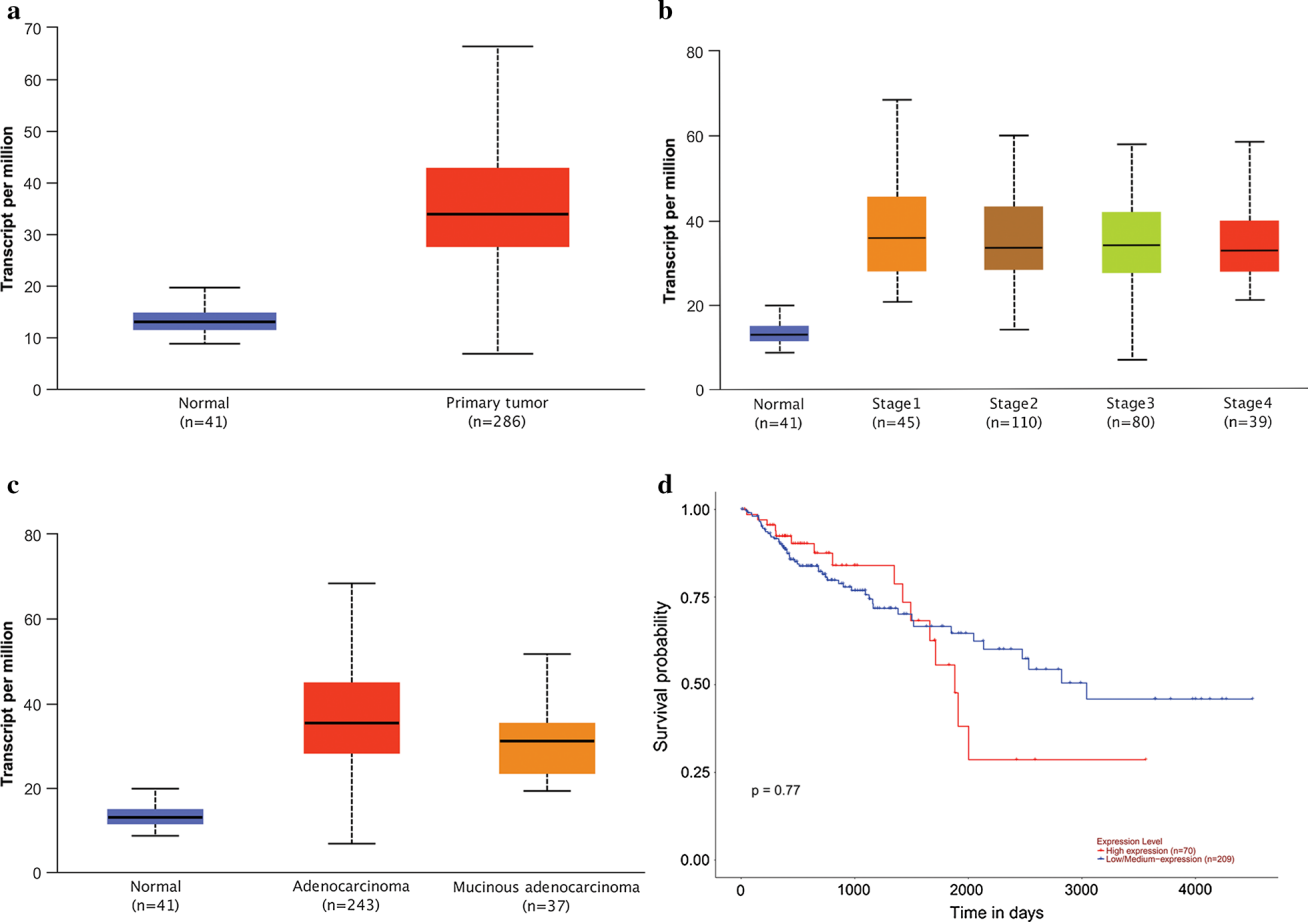
WDR43 is associated with the stage and subtype of CRC. TCGA suggested that WDR43 is **a** upregulated and correlated with **b** disease stage and **c** tumor subtype, but not with **d** prognosis. *p* < 0.05. *WDR* WD40 repeat, *CRC* colorectal cancer, *TCGA* The Cancer Genome Atlas

Following a search of the TCGA database, the expression of WDR43 was found to be increased in CRC, as compared to that in cancer-adjacent normal colon tissue (Fig. 1a). The upregulation of WDR43 was found to be associated with the stage (Fig. 1b) and subtype (Fig. 1c) of CRC, but exhibited no significant correlation with prognosis (Fig. 1d).

### WDR43 expression is increased in CRC tissues

**Fig. 2:**
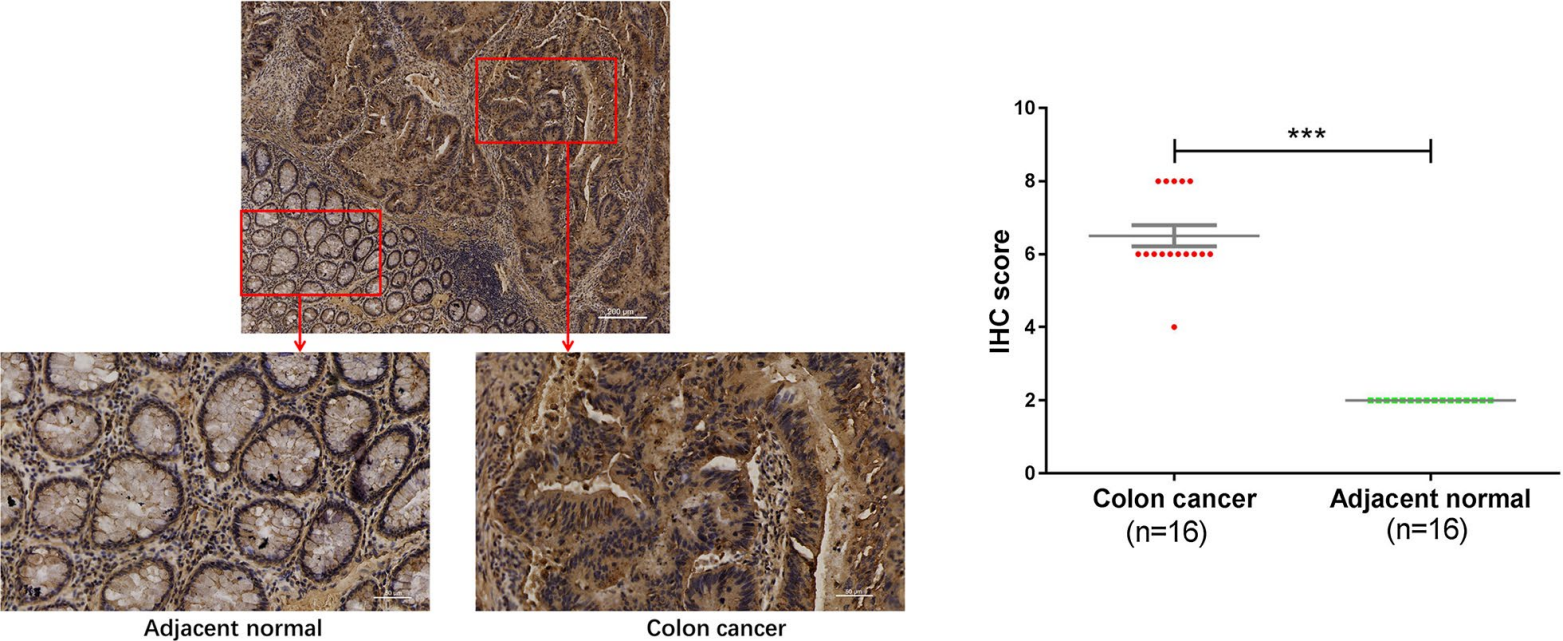
WDR43 expression is increased in CRC tissues. Representative images of immunohistochemical staining for WDR43 in tissue microarrays. ***p < 0.001. Magnification ×50. *WDR* WD40 repeat, *CRC* colorectal cancer

Immunohistochemistry was used to examine 16 pairs of cancer specimens and normal tissues adjacent to cancer from CRC patients. The immunohistochemical score (% of positive cells × staining intensity) showed that the expression level of WDR43 in CRC tumor tissue was higher compared with that cancer-adjacent normal tissues (Fig. 2).

### WDR43 is highly expressed in CRC cells

**Fig. 3:**
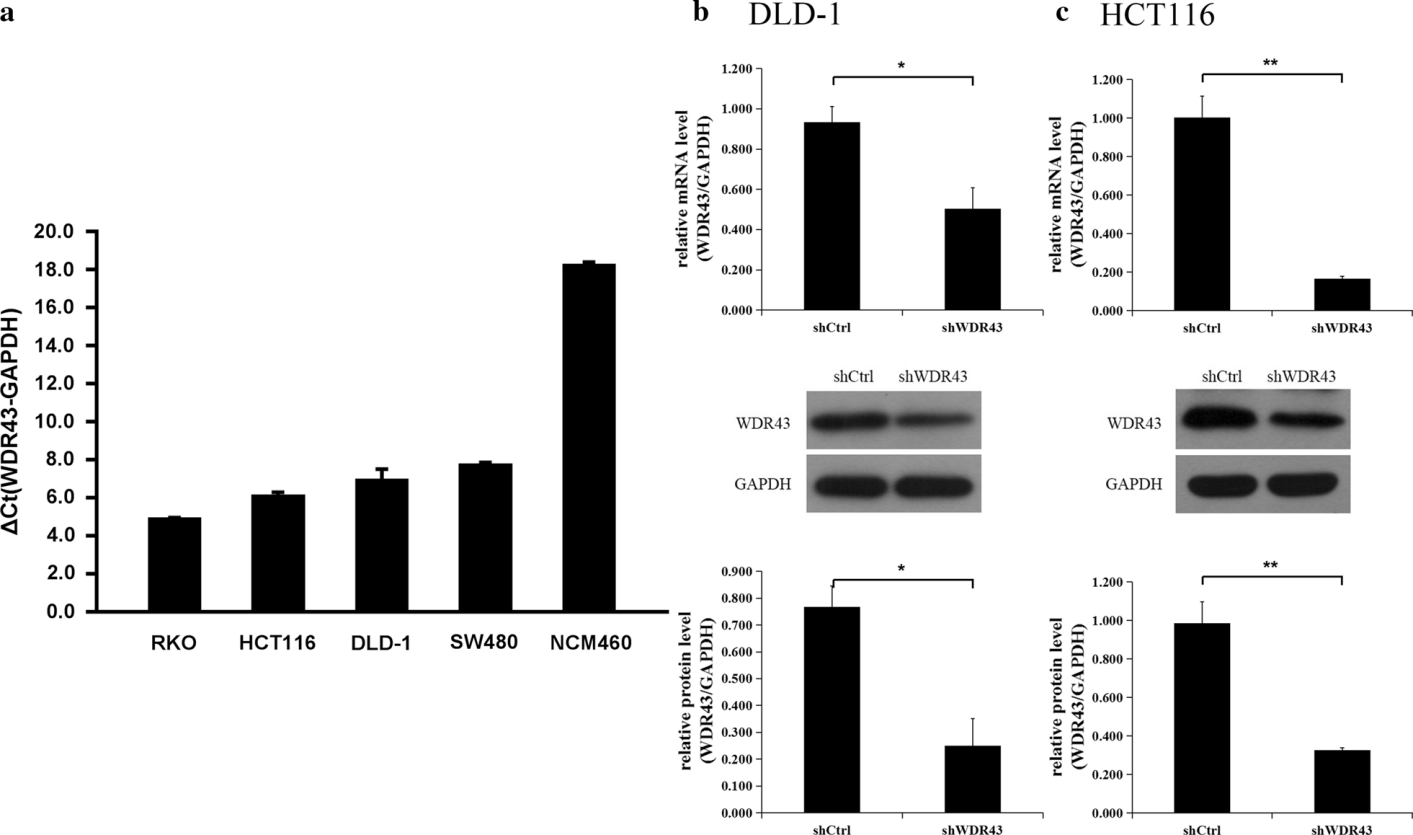
WDR43 is highly expressed in CRC cells. **a** WDR43 mRNA expression levels in a normal colonic cell line and 4 CRC cell lines. Gene and protein levels of WDR43 in **b** DLD-1 and **c** HCT116 cells transfected with lentiviral targeting WDR43. *p < 0.05, **p < 0.01. *WDR* WD40 repeat, *CRC* colorectal cancer, shCtrl negative control, *shWDR43* cells transfected with lentiviral targeting WDR43

The level of WDR43 mRNA was detected in 4 CRC cell lines and the NCM460 human normal colonic epithelial cell line (Fig. 3a). As compared with NCM460, the WDR43 expression was increased in the 4 CRC cell lines. To reduce contingency, two cell lines with a medium expression level were selected for subsequent functional tests. Lentivirus was used to obtain DLD-1 and HCT116 cells with stably knocked down WDR43. RT-qPCR and western blotting (WB) were performed to evaluate WDR43 knockdown and confirm the knockdown efficiency in the two cell lines (Fig. 3b, c, Additional file 2: Figure S1).

### WDR43 knockdown inhibits the proliferation and colony formation of CRC cells in vitro

**Fig. 4:**
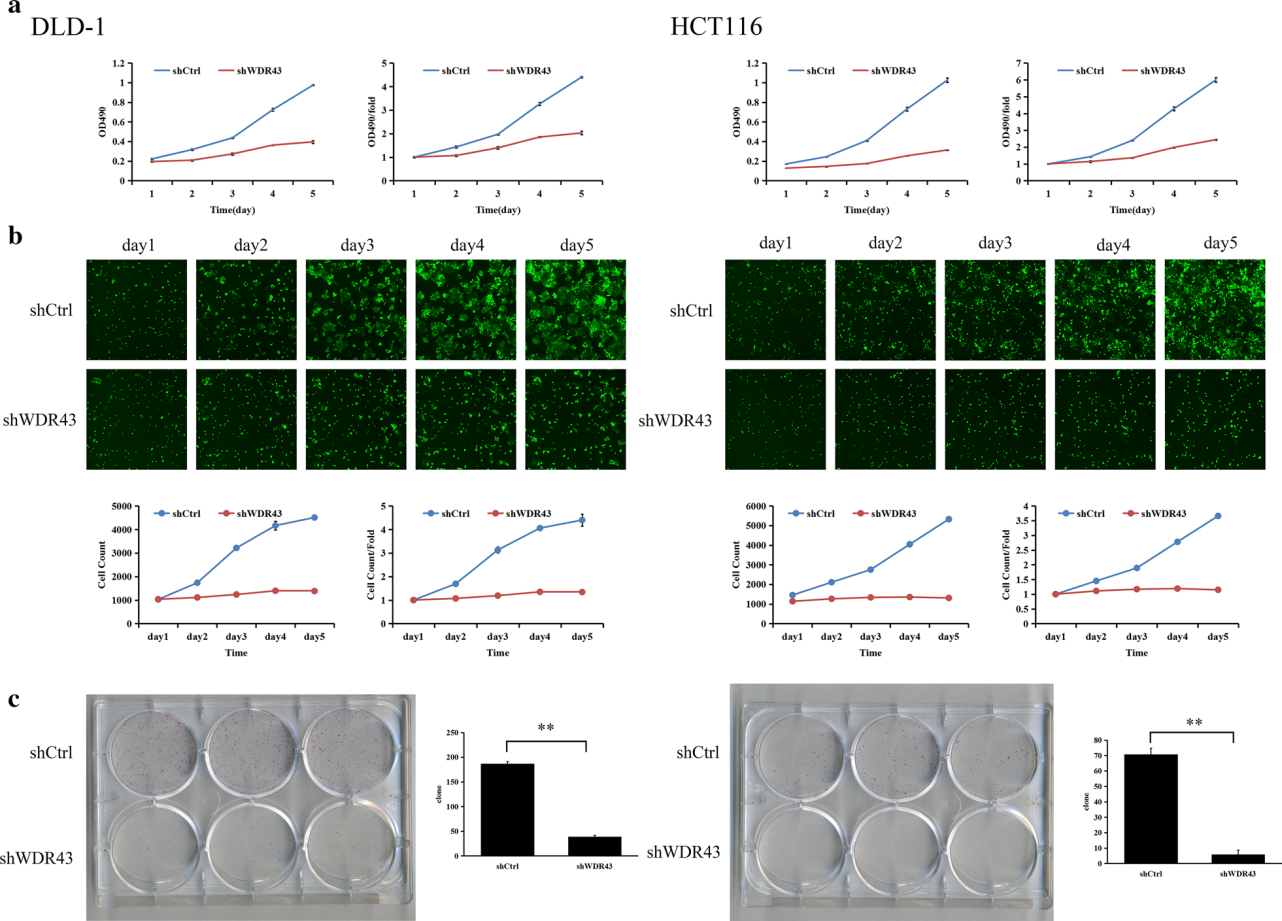
WDR43 knockdown inhibits the proliferation and colony formation of CRC cells in vitro. Cell proliferation was detected for five consecutive days using **a** MTT and **b** Celigo assays in DLD-1 (the left half of the figure) and HCT116 (the right half of the figure) cells, respectively (statistically significant differences of proliferation in two assays were found in day 4 and day 5, *p* < 0.05). **c** Colony formation assays were performed in both DLD-1(the left half of the figure) and HCT116 cells(the right half of the figure). **p < 0.01. *WDR* WD40 repeat, *CRC* colorectal cancer, *shCtrl* negative control, *shWDR43* cells transfected with lentiviral targeting WDR43

Following WDR43 knockdown, the proliferative ability of the DLD-1 and HCT116 cell lines was examined. The cells were continuously counted by MTT (Additional file 3: Table S1) and Celigo (Additional file 4: Table S2) experiments for 5 days. It was found that, following WDR43 knockdown, the proliferative rate of DLD-1 and HCT116 cells was significantly inhibited (Fig. 4a, b, Additional file 5: Figure S2), suggesting that low WDR43 expression inhibits CRC cell proliferation. The colony formation experiment also confirmed this result (Fig. 4c).

### WDR43 knockdown promotes apoptosis in CRC cells

**Fig. 5:**
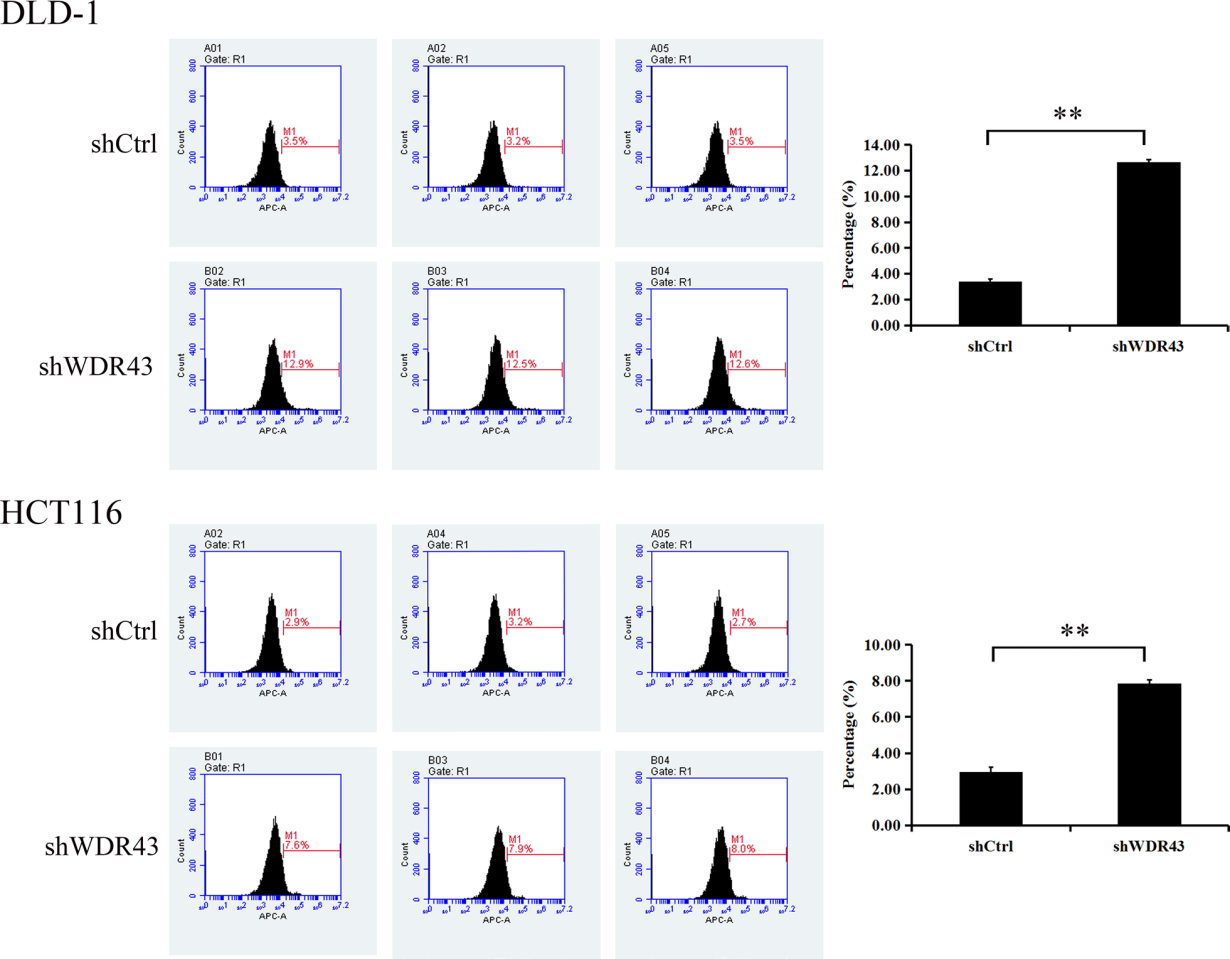
WDR43 knockdown promotes the apoptosis of CRC cells. The percentage of apoptotic cells was demonstrated by flow cytometry in DLD-1 and HCT116 cell lines following WDR43 knockdown. **p < 0.01. *WDR* WD40 repeat, *CRC* colorectal cancer, shCtrl negative control, *shWDR43* cells transfected with lentiviral targeting WDR43

Flow cytometry was performed to investigate whether WDR43 promotes CRC cell proliferation by regulating apoptosis. It was shown that changes in WDR43 levels affect the apoptotic rate. As compared with the control group, the number of apoptotic cells increased in the two cell lines with a decreased WDR43 gene expression (Fig. 5, Additional file 6: Table S3 and Additional file 7: Table S4).

### WDR43 knockdown inhibits migration and invasion in CRC cells

**Fig. 6:**
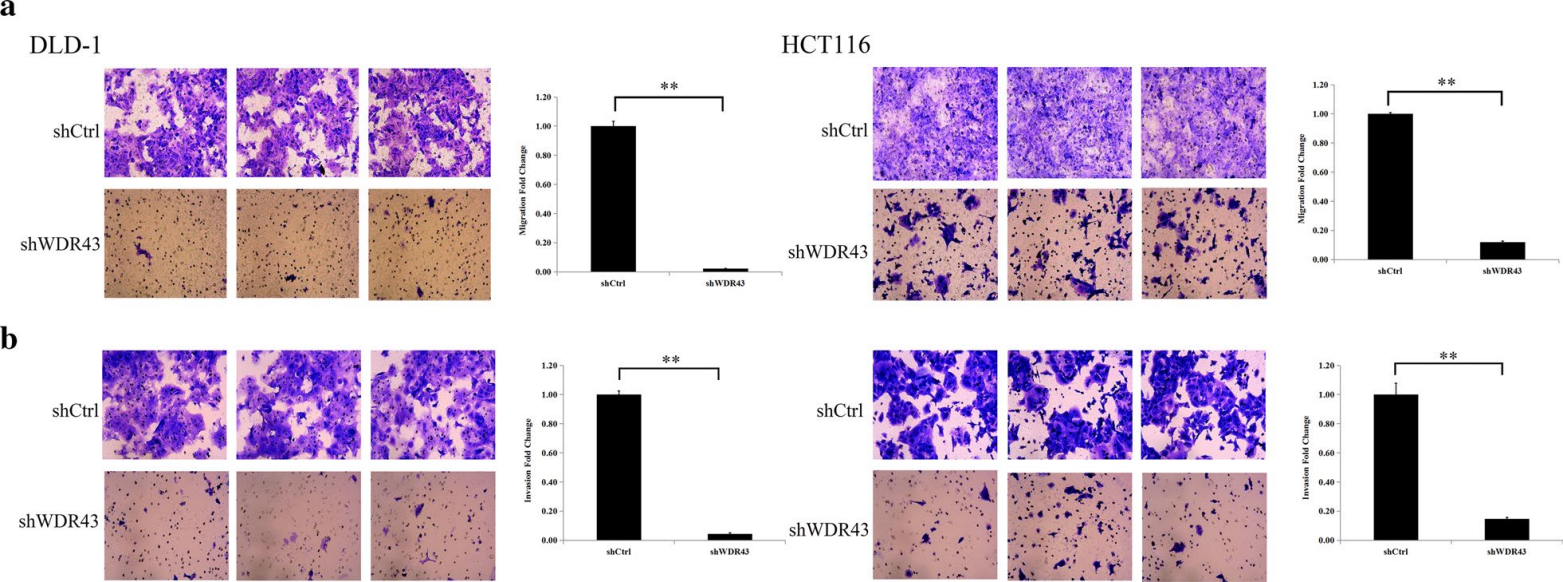
WDR43 knockdown inhibits the migration and invasion of CRC cells. WDR43 knockdown suppressed both **a** migration and **b** invasion in DLD-1 (the left half of the figure) and HCT116 cells (the right half of the figure), as shown by Transwell migration and invasion assays, respectively. **p < 0.01. *WDR* WD40 repeat, *CRC* colorectal cancer, shCtrl negative control, *shWDR43* cells transfected with lentiviral targeting WDR43

Transwell assay was performed to investigate the effect of WDR43 knockdown on the invasion and migration of CRC cells. After 24 and 48 h of viral infection, the migrating and invasive cells were counted, and it was found that WDR43 knockdown inhibited the migration and invasion of CRC cells (Fig. 6a, b, Additional file 8: Figure S3).

### WDR43 knockdown suppresses vimentin (VIM) expression

**Fig. 7:**
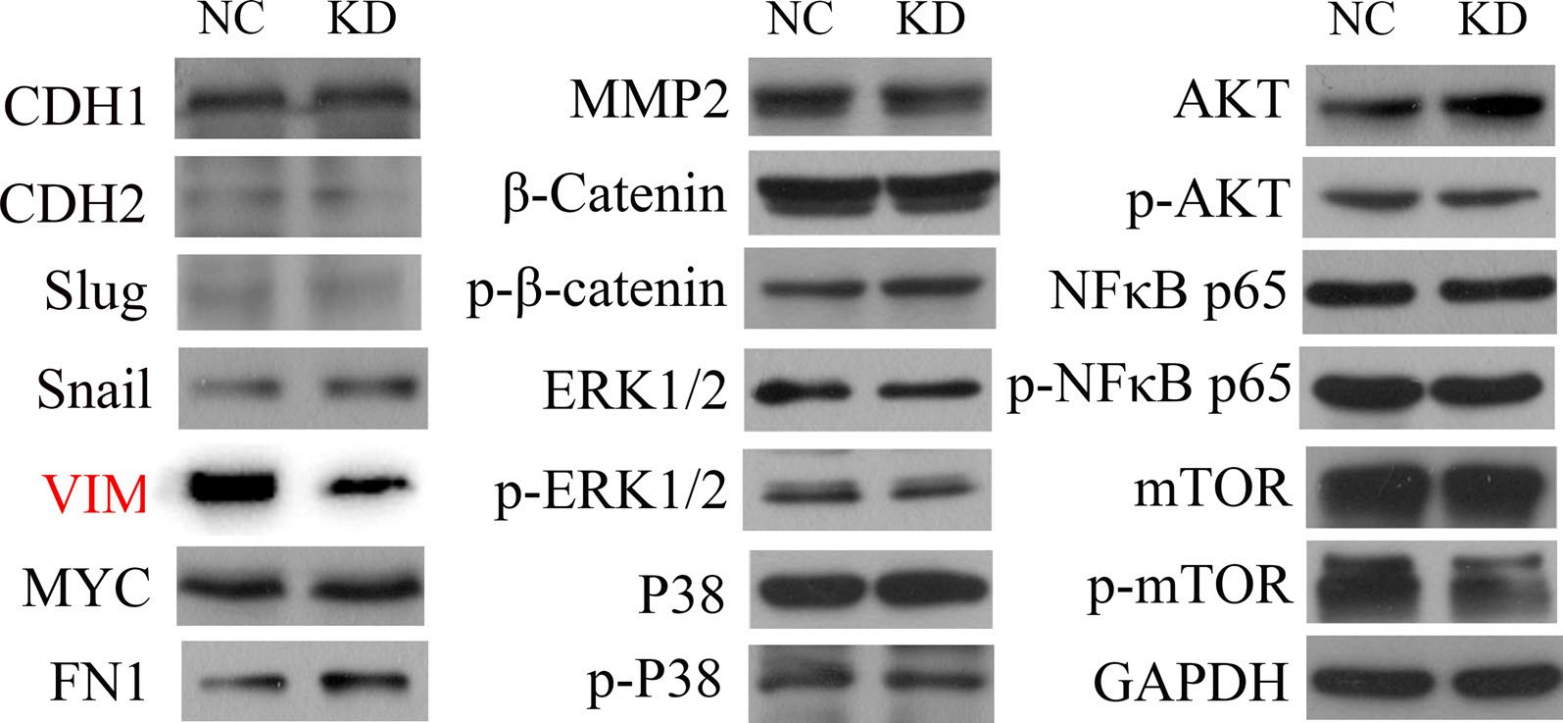
WDR43 knockdown suppresses VIM expression. The levels of several proteins associated with tumor cell proliferation and migration as well as their phosphorylated forms were examined by western blotting in DLD-1 cells, as were those of classical signaling pathway proteins. *CDH1* E-cadherin, *CDH2* N-cadherin, *FN1* fibronectin 1, *(p-)AKT* (phosphorylated-)protein kinase B, *MMP-2* matrix metalloproteinase-2, *(p-)mTOR* (phosphorylated-)mechanistic target of rapamycin, *(p-)NF-κB p65* (phosphorylated-)nuclear factor-κB p65, *(p-)ERK1/2* (phosphorylated-)extracellular signal-regulated kinase1/2; *VIM* vimentin, *NC* negative control, *KD* WDR43 knockdown

To explore the mechanism underlying the effect of WDR43 on the proliferation and migration of CRC cells, the downstream functional proteins that may be regulated by WDR43 were examined. Following the lentiviral knockdown of WDR43 in DLD-1 cells, WB was used to detect the expression of several proteins associated with tumor cell proliferation and migration, including N-cadherin, E-cadherin, Slug, Snail, VIM, MYC, fibronectin 1 and matrix metalloproteinase-2, and classic signaling pathway proteins and their phosphorylated forms, including β-catenin, p-β-catenin, extracellular signal-regulated kinase (ERK)1/2, p-ERK1/2, P-38, p-P38, protein kinase B (Akt), p-Akt, nuclear factor-κB (NF-κB) p65, p-NF-κB p65, mechanistic target of rapamycin (mTOR) and p-mTOR. The results showed that, following WDR43 knockdown, the expression of VIM was significantly reduced (Fig. 7).

### VIM overexpression partially restores the proliferative and migratory ability of CRC cells following WDR43 knockdown

**Fig. 8:**
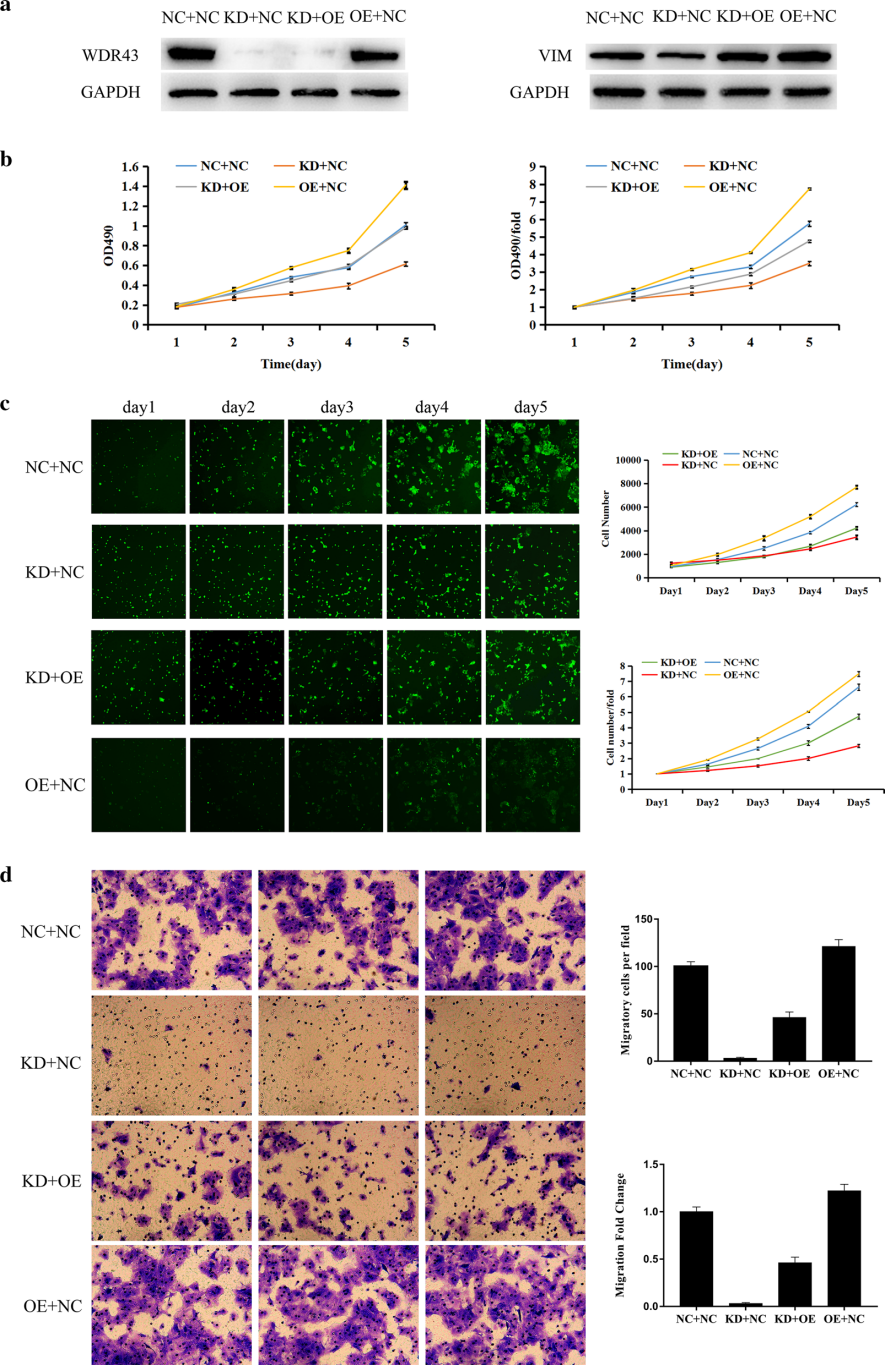
VIM overexpression partially restores the proliferative and migratory ability of CRC cells following WDR43 knockdown. **a** The expression of WDR43 and VIM in each group were detected by western blotting. Cell proliferation was detected by **b** MTT and **c** Celigo assays in DLD-1 cells (statistically significant differences of proliferation between KD + NC and NC + NC, KD + OE and KD + NC, OE + NC and KD + OE in two assays were found in day 4 and day 5, *p* < 0.05). **d** Transwell migration assay was also performed in DLD-1 cells (statistically significant differences of migration between KD + NC and NC + NC, KD + OE and KD + NC, OE + NC and KD + OE in two assays were found, *p* < 0.05). *VIM* vimentin, *CRC* colorectal cancer, *WDR* WD40 repeat, *NC* negative control, *KD* WDR43 knockdown, *OE* VIM overexpression

To confirm the function of VIM, lentivirus was used to simultaneously knock down WDR43 and overexpress VIM in DLD-1 cells. WB was used to test the transfection efficiency and stably transfected strains were obtained (Fig. 8a). MTT and Celigo experiments were used to test the proliferative ability of the cells and found that, following VIM overexpression, the suppression of cell proliferation caused by WDR43 knockdown was significantly recovered, while tumor cells overexpressing VIM alone showed stronger proliferative ability (Fig. 8b, c; Additional file 9: Table S5 and Additional file 10: Table S6). Transwell assay results also showed that, as compared with two control groups, the migration of DLD-1 cells with WDR43 knockdown and VIM overexpression were intermediate (Fig. 8d).

### WDR43 knockdown inhibits the proliferation of CRC cells in vivo

**Fig. 9:**
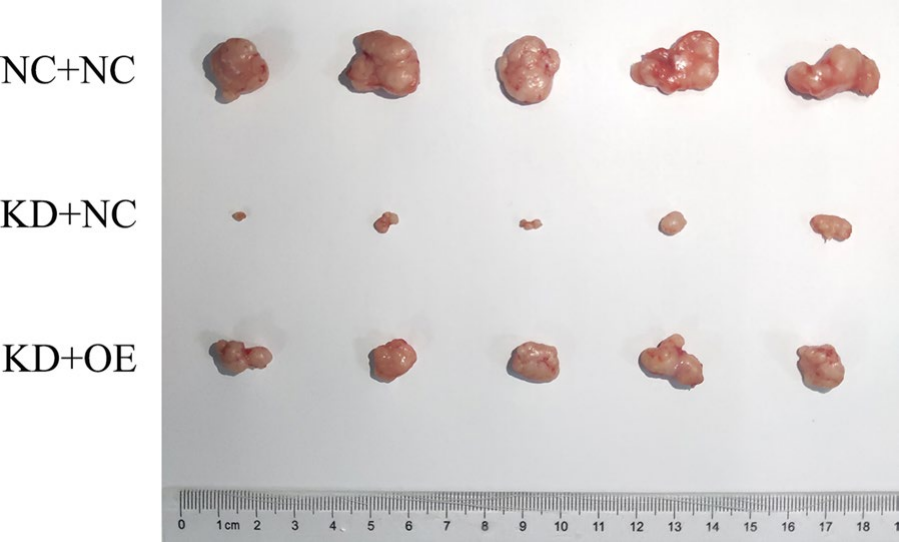
WDR43 knockdown inhibits the proliferation of CRC cells in vivo. Images of subcutaneous xenografts from mice in the WDR43 knockdown, WDR43 knockdown with VIM overexpression, and NC groups. *n* = 5. *WDR* WD40 repeat, *CRC* colorectal cancer, *VIM* vimentin, *NC* negative control, *KD* WDR43 knockdown, *OE* VIM overexpression

To study whether WDR43 plays a same role in the proliferation of CRC in vivo, a subcutaneous xenograft model of Balb/c nude mice was established using DLD-1 cells. As compared with the control group, the tumor volume and weight of mice in the WDR43 knockdown group was reduced, while the tumor volume and weight of mice with both WDR43 knockdown and VIM overexpression were significantly recovered (Fig. 9). Therefore, a low WDR43 expression decreases the growth of tumor in vivo, which was accord with our research in vitro and clinical discoveries.

## Discussion

In the present study, high WDR43 expression level was confirmed in CRC cells and tissues, and it was found that WDR43 promotes the proliferation, migratory, invasive and anti-apoptotic ability of CRC cells. At the same time, WDR43 was also found to regulate the expression of downstream functional protein VIM. These results preliminarily illustrated the role of WDR43 in the development of CRC, and may provide a valuable reference for further expanding the application of WDR43 in tumor research.

As one of the most plentiful domains of mankind, the WDR domain has been shown to participate in a wide range of cellular networks [7]. This domain has been associated with several human diseases [6, 7]. Particularly in the field of oncology, certain proteins containing WDR domains may be used as effective targets for cancer treatment [13]. Grebien et al. [14] used the specific WDR5 antagonist OICR-9429 to disrupt the MLL1-WDR5 interaction, thereby killing p30-expressing acute myeloid leukemia cells. O’Bryant et al. [15] found that WDR77 knockdown can inhibit E2F3 activation and enhance TGFβ signaling, abolishing the prostate tumorigenesis induced by PTEN deletion. Based on the correlation between WDR protein and cancer, CRC-related genes were screened out by genome-wide gene expression profiling [16], and WDR43 was selected to explore its impact on tumor biological functions. In the present study, the inhibitory effect of WDR43 knockdown on CRC in vivo and in vitro was observed. Similarly, it was found by Zhao et al. [17] that the loss of WDR43 in zebrafish caused early developmental defects in various tissues. In addition, Fujimura et al. [18] proved that the nucleolar protein 11 (NOL11) forms a protein complex called NWC with WDR43 and Cirhin, and participates in the process of chromosome mitosis by promoting the enrichment of Aurora B on the centromere. The present study also reported an effect of WDR43 on the migratory ability of CRC cells, an effect that appeared to be achieved through the regulation of VIM, which was manifested as a significant decrease in VIM expression following WDR43 knockdown. Similarly, in the study by Zhu et al. [19], miR-34a inhibited the expression of VIM and reduced the migratory and invasive ability of CRC cells. As the main component of the intermediate filament protein family, VIM has the ability to maintain cell integrity and enable resistance to stress [20]. A number of studies have confirmed that increased expression of VIM is associated with tumor metastasis and invasiveness [21], and is considered to be a classic marker of epithelial–mesenchymal transition (EMT) [22]. Although the effect of overexpression of VIM on the proliferation and migration of CRC cells was confirmed, the evidence obtained was insufficient to support the involvement of EMT in the WDR43 regulatory network, and further research is necessary. Finally, with regards to the failure of this study to screen out the specific signaling pathways that WDR43 may directly regulate, the possible explanation is that WDR43 may play a more basic role in nucleic acid metabolism, which requires the support of bioinformatics and subsequent deeper mining.

## Conclusion

In conclusion, to the best of our knowledge, this study is the first to report that WDR43 knockdown inhibits the proliferative, migratory, invasive and anti-apoptotic ability of CRC cells. These findings have enriched our understanding of and research on WDR domains, providing a valuable reference for the diagnosis and treatment of CRC in the future.

## Supplementary Information

**Additional file 1.** The sequence of VIM overexpression.

**Additional file 2: Figure S1**. Protein levels of WDR43 in DLD-1 cells transfected with another lentiviral targeting WDR43.

**Additional file 3: Table S1.** Using MTT assays to detect OD490 and OD490/fold of DLD-1 and HCT116 cells and corresponding shWDR43 cell lines.

**Additional file 4: Table S2.** Using Celigo assays to detect cell count and cell count/fold of DLD-1 and HCT116 cells and corresponding shWDR43 cell lines.

**Additional file 5: Figure S2.** Cell proliferation was detected in DLD-1 cells for 5 consecutive days using Celigo assays.

**Additional file 6: Table S3.** Apoptosis of DLD-1 cells detected by flow cytometry.

**Additional file 7: Table S4.** Apoptosis of HCT116 cells detected by flow cytometry.

**Additional file 8: Figure S3.** Invasiveness of the DLD-1 cells was detected by transwell invasion assays.

**Additional file 9: Table S5.** Using MTT assays to detect OD490 and OD490/fold of DLD-1 cell line and corresponding shWDR43 and VIM-overexpression cell line.

**Additional file 10: Table S6.** Using Celigo assays to detect cell count and cell count/fold of DLD-1 cell line and corresponding shWDR43 and VIM-overexpression cell line.

## Data Availability

The data sets analysed during the current study are available in the TCGA repository (http://ualcan.path.uab.edu/cgi-bin/TCGAExResultNew2.pl?genenam=WDR43&ctype=COAD).

## Abbreviations

WDR43: WD40 repeat 43
CRC: Colorectal cancer
VIM: Vimentin
18S rRNA: Small-subunit ribosomal RNA
TMAs: Tissue microarrays
RT-qPCR: Reverse transcription quantitative polymerase chain reaction
WB: Western blotting
CDH1: E-cadherin
CDH2: N-cadherin
FN1: Fibronectin 1
(p-)AKT: (Phosphorylated-)protein kinase B
MMP-2: Matrix metalloproteinase-2
(p-)mTOR: (Phosphorylated-)mechanistic target of rapamycin
(p-)NF-κB p65: (Phosphorylated-)nuclear factor-κB p65
(p-)ERK1/2: (Phosphorylated-)extracellular signal-regulated kinase1/2
VIM: Vimentin
NC: Negative control
KD: WDR43 knockdown
OE: VIM overexpression
EMT: Epithelial–mesenchymal transition
