# Supplementary material for: WD40 repeat 43 mediates cell survival, proliferation, migration and invasion via vimentin in colorectal cancer
Source: Cancer Cell Int. 2021 Aug 9;21:418. doi: 10.1186/s12935-021-02109-1 (PMC8351096; doi:10.1186/s12935-021-02109-1)
Supplement: Supplementary file 1 — Additional file 1. The sequence of VIM overexpression. [file 12935_2021_2109_MOESM1_ESM.docx]

**The sequence of VIM overexpression**

atgtccacc aggtccgtgt cctcgtcctc ctaccgcagg atgttcggcg gcccgggcac cgcgagccgg ccgagctcca gccggagcta cgtgactacg tccacccgca cctacagcct gggcagcgcg ctgcgcccca gcaccagccg cagcctctac gcctcgtccc cgggcggcgt gtatgccacg cgctcctctg ccgtgcgcct gcggagcagc gtgcccgggg tgcggctcct gcaggactcg gtggacttct cgctggccga cgccatcaac accgagttca agaacacccg caccaacgag aaggtggagc tgcaggagct gaatgaccgc ttcgccaact acatcgacaa ggtgcgcttc ctggagcagc agaataagat cctgctggcc gagctcgagc agctcaaggg ccaaggcaag tcgcgcctgg gggacctcta cgaggaggag atgcgggagc tgcgccggca ggtggaccag ctaaccaacg acaaagcccg cgtcgaggtg gagcgcgaca acctggccga ggacatcatg cgcctccggg agaaattgca ggaggagatg cttcagagag aggaagccga aaacaccctg caatctttca gacaggatgt tgacaatgcg tctctggcac gtcttgacct tgaacgcaaa gtggaatctt tgcaagaaga gattgccttt ttgaagaaac tccacgaaga ggaaatccag gagctgcagg ctcagattca ggaacagcat gtccaaatcg atgtggatgt ttccaagcct gacctcacgg ctgccctgcg tgacgtacgt cagcaatatg aaagtgtggc tgccaagaac ctgcaggagg cagaagaatg gtacaaatcc aagtttgctg acctctctga ggctgccaac cggaacaatg acgccctgcg ccaggcaaag caggagtcca ctgagtaccg gagacaggtg cagtccctca cctgtgaagt ggatgccctt aaaggaacca atgagtccct ggaacgccag atgcgtgaaa tggaagagaa ctttgccgtt gaagctgcta actaccaaga cactattggc cgcctgcagg atgagattca gaatatgaag gaggaaatgg ctcgtcacct tcgtgaatac caagacctgc tcaatgttaa gatggccctt gacattgaga ttgccaccta caggaagctg ctggaaggcg aggagagcag gatttctctg cctcttccaa acttttcctc cctgaacctg agggaaacta atctggattc actccctctg gttgataccc actcaaaaag gacacttctg attaagacgg ttgaaactag agatggacag gttatcaacg aaacttctca gcatcacgat gaccttgaat aa
