# Supplementary figures and images for: WD40 repeat 43 mediates cell survival, proliferation, migration and invasion via vimentin in colorectal cancer
Source: Cancer Cell Int. 2021 Aug 9;21:418. doi: 10.1186/s12935-021-02109-1 (PMC8351096; doi:10.1186/s12935-021-02109-1)

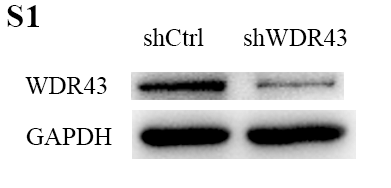

Supplement: Supplementary file 2 — Additional file 2: Figure S1. Protein levels of WDR43 in DLD-1 cells transfected with another lentiviral targeting WDR43. [file 12935_2021_2109_MOESM2_ESM.tif]

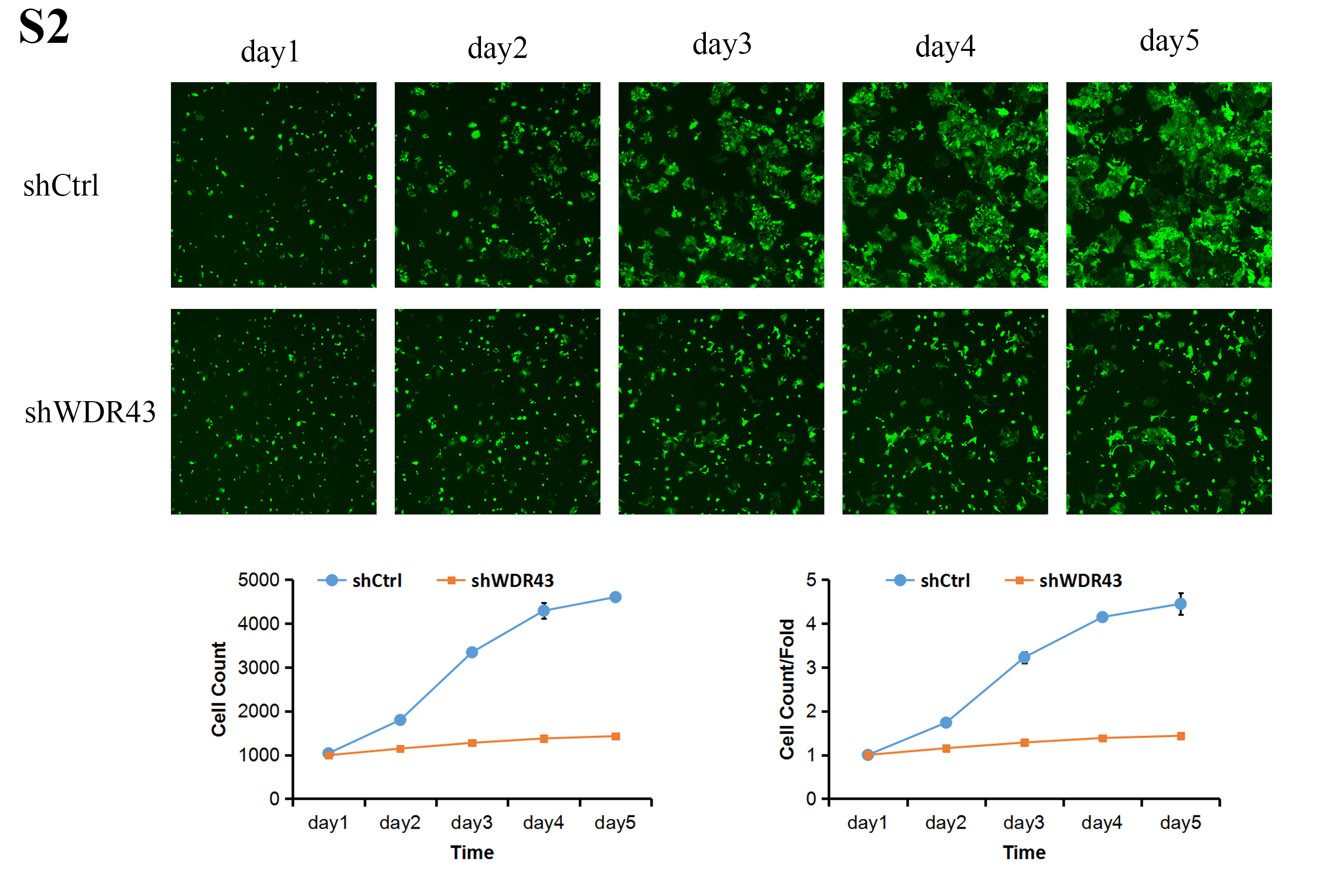

Supplement: Supplementary file 5 — Additional file 5: Figure S2. Cell proliferation was detected in DLD-1 cells for 5 consecutive days using Celigo assays. [file 12935_2021_2109_MOESM5_ESM.tif]

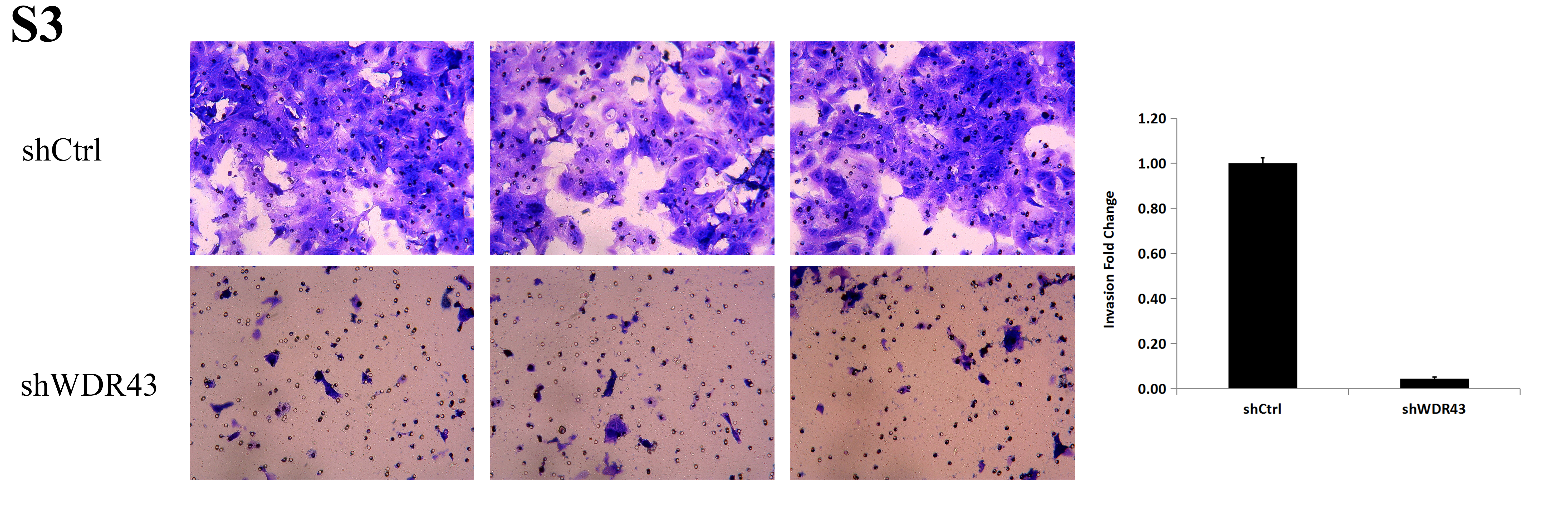

Supplement: Supplementary file 8 — Additional file 8: Figure S3. Invasiveness of the DLD-1 cells was detected by transwell invasion assays. [file 12935_2021_2109_MOESM8_ESM.tif]
